# Modular metamaterials composed of foldable obelisk-like units with reprogrammable mechanical behaviors based on multistability

**DOI:** 10.1038/s41598-019-55222-7

**Published:** 2019-12-11

**Authors:** Nan Yang, Mingkai Zhang, Rui Zhu, Xiao-dong Niu

**Affiliations:** 1 Intelligent Manufacturing Key Laboratory of Ministry of Education, Shantou University, Shantou, 515063 China; 2 Key Laboratory of Dynamics and Control of Flight Vehicle, Ministry of Education, School of Aerospace Engineering, Beijing Institute of Technology, Beijing, 100081 China

**Keywords:** Structure of solids and liquids, Mechanical engineering

## Abstract

A new type of modular metamaterials with reprogrammable mechanical properties is proposed based on the multistability in decoupled units. This metamaterial consists of periodically arranged foldable obelisk-like (FO) units, and each unit has three interchangeable states: two different soft states and a stiff state. Therefore, such metamaterial can possess various mechanical properties with different state combinations of units. Both theoretical and experimental investigations are conducted to understand the multistability in one unit and the reprogrammed mechanical properties in a two-dimensional tessellation. Additionally, we investigate the inverse question that whether the identical force response can be generated with different geometrical design of the metamaterial and propose a way to build 3D metamaterials with intended architectures. This work establishes general principles for designing mechanical metamaterials with independently transformable modules, and opens new avenues for various potential applications such as: self-locking materials, impact mitigation and stiffness transformation materials.

## Introduction

Recently, metamaterials with unusual emergent properties, such as negative material properties (negative Poisson’s ratio, negative refractive index, etc.) or multistability, and with untraditional design concepts have gained enormous attentions among scientists and engineers^1–9^. Origami-inspired techniques with the ability to design and fabricate customizable and responsive mechanical metamaterials certainly become popular^10–12^. A variety of natural systems already show origami patterns, such as wings^13^, leaves^14^, and flower petals^15^. In the artificial counterparts, ranging from solar sails^16^, space mirrors, aircraft wings, and robots^17^, to microporous devices^18^, meta-surface^19^, artificial swings^20^ and programmed self-assembly of nucleic acid strands^21^, the foldable structures can be realized based on origami-inspired techniques. For general origami structures, there’re kinematic compatibility constraints between units^22–26^, which makes it difficult to generate desire geometrical patterns and further limits the variety of mechanical properties. Nevertheless, to weaken the kinematic constraints, current methods are allowing bending on facets^10,11,22^ and designing interchangeable modular in cellular structures^27^. Here, we design a new kind of modular metastructures, which naturally have independently transformable unit cells and therefore, can possess unique reprogrammable mechanical properties in 2D/3D assemblies.

## Results and Discussion

### Geometry and mechanics of a FO unit

**Figure 1:**
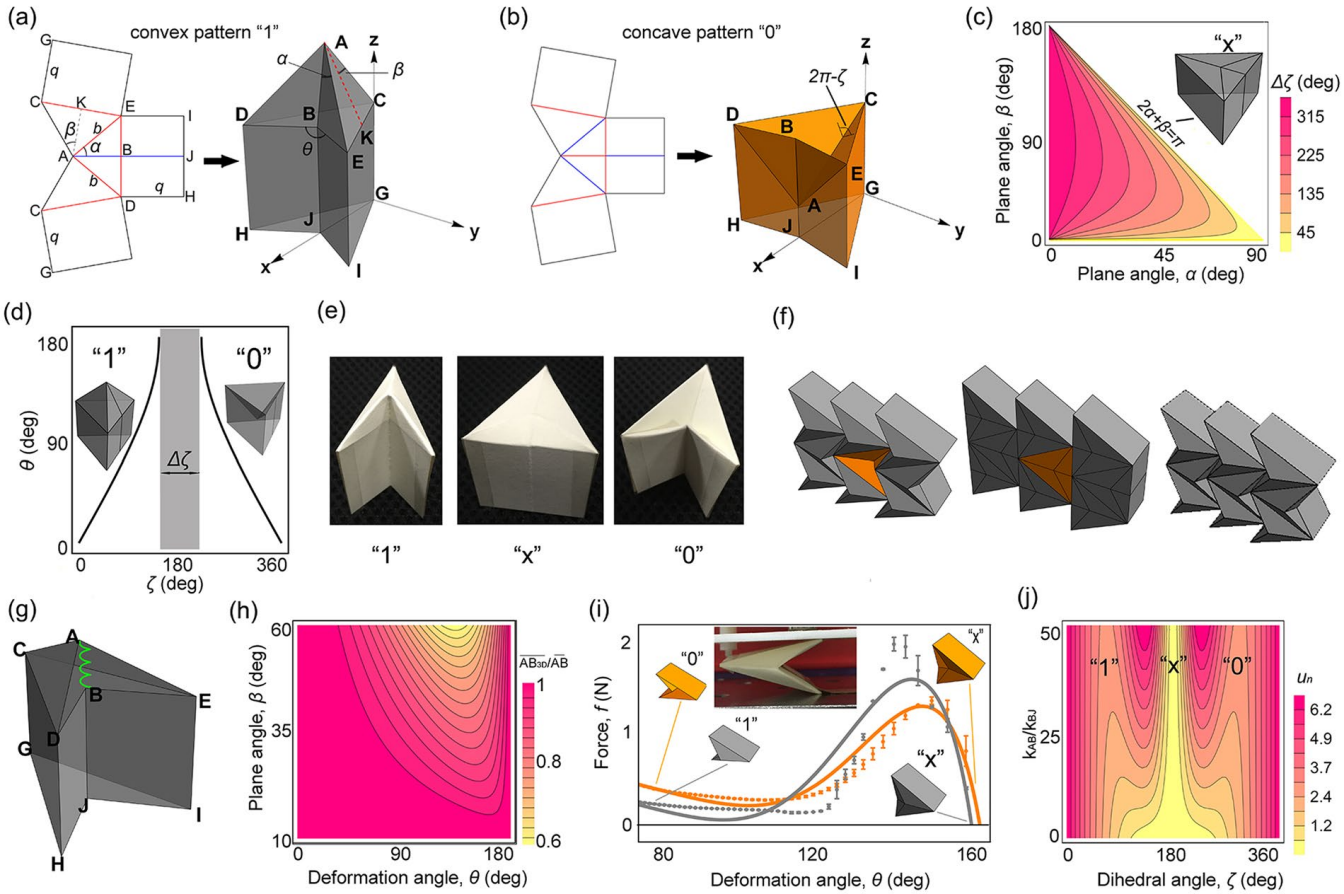
Unit geometry and mechanical property. (**a**) 2D folding pattern and 3D convex pattern “1” (red: mountain creases, blue: valley creases), the plane angles $\alpha =\angle {\rm{BAE}}=\angle {\rm{KAE}},\beta =\angle {\rm{CAK}}$, the lengths $b=\overline{{\rm{AE}}}=\overline{{\rm{AD}}}$ and $q=\overline{{\rm{CG}}}=\overline{{\rm{DH}}}=\overline{{\rm{BJ}}}=\overline{{\rm{EI}}}$, the deformation angle *θ* defined as the dihedral angle between facets BDHJ and BEIJ, (**b**) 2D folding pattern and 3D concave pattern “0”, the angle *ζ* defined as the dihedral angle between facets ACD and ACE (in pattern “1” $\zeta  < 180^\circ$, and in pattern “0” $\zeta  > 180^\circ$), (**c**) $\Delta \zeta$ as a function of *α* and *β* (the 3D unit with $\theta =180^\circ$ and $2\alpha +\beta =180^\circ$ shows pattern “x”), (**d**) the relationship between *θ* and *ζ* and the definition of $\Delta \zeta$ in the gray region (here two 3D units with $\alpha =\beta =40^\circ$ in patterns “1” and “0”), (**e**) photographs of single unit in state “1”, “x”, and “0”. (**f**) One unit in a 2D tessellation independently switches from pattern “0” to “1” (via “x”). (**g**) The spring model, facets ABD and ABE are replaced with a green virtual spring and crease BJ is assumed as a torsional spring, (**h**) $\overline{{{\rm{AB}}}_{3{\rm{D}}}}/\overline{{\rm{AB}}}$ as a function of *θ* and *β* with $\alpha =60^\circ$, (**i**) Force experimental data with error bars VS. the simulated curves for the FO unit with $\alpha =\beta =60^\circ$ and $b=q=20\,{\rm{mm}}$, gray and orange denote “1” and “0” pattern (insert shows the experimental setup). (**j**) Normalized energy *u*_*n*_ as a function of *ζ* and ${k}_{{\rm{AB}}}/{k}_{{\rm{BJ}}}$, with three local minima (patterns “1”, “x”, and “0”).

Figure 1a,b show the 2D folding patterns and their corresponding 3D units. Particularly, two 3D patterns can be formed: a convex pattern (state “1”) is obtained by setting creases AE, AB and AD as mountain, valley and mountain crease, respectively (Fig. 1a); a concave pattern (state “0”) is obtained by setting AE, AB and AD as valley, mountain and valley crease (Fig. 1b). There are five parameters, plane angles *α*, *β* and lengths *b*, *q* and deformation angle, which control the 3D configurations of the FO unit. Additionally, an angle *ζ* is defined as the dihedral angle between facets EAC and DAC. In pattern “1” (Fig. 1a), $\zeta  < 180^\circ$. In pattern “0” (Fig. 1b), $\zeta  > 180^\circ$. We define $\Delta \zeta$ as the degree of difficulty for pattern “1” switching to pattern “0” as $\Delta \zeta =|{\zeta }_{\theta =180^\circ }^{1}-{\zeta }_{\theta =180^\circ }^{0}|$, where ${\zeta }_{\theta =180^\circ }^{1}$ and ${\zeta }_{\theta =180^\circ }^{0}$ denote angle *ζ* with $\theta =180^\circ$ in pattern “1” and pattern “0”, respectively.

As shown in Fig. 1c, the switch from pattern “1” to pattern “0” is very difficult due to the high value of $\Delta \zeta$ for $2\alpha +\beta  < 180^\circ$ while the switch becomes much easier along the diagonal with $\Delta \zeta =0$ where $2\alpha +\beta =180^\circ$. Here, we define pattern “x” (where $\theta =\zeta =180^\circ$, see insert for the 3D example) as an important transformation between patterns “1” and “0”. By plotting the $\zeta -\theta$ relationship with $2\alpha +\beta  < 180^\circ$ in Fig. 1d, it can be clearly seen that the transformation between pattern “1” and “0” (see inserts) is impossible due to $\Delta \zeta  > 0$. The overall geometry and $\Delta \zeta$ can be found in the Supporting Information (SI) Section 1 and 2. Therefore, to make the transformation feasible, a unit with $2\alpha +\beta =180^\circ$ $(\alpha =\beta =60^\circ )$, is made with its photographs in three patterns being shown in Fig. 1e. More interestingly, such a unit has the advantage of independently changing its pattern in a 2D cellular structure. As shown in Fig. 1f, if one intends to switch the central orange unit from pattern “0” to “1” while not change the surrounding gray units, then every unit can first transform into the pattern “x” (the middle part of Fig. 1f) and then, achieve its own final state, as shown in the right part of Fig. 1f. To switch each unit between the convex pattern (Fig. 1a) and the concave pattern (Fig. 1b) in a cellular structure (Fig. 1f), we could control the angle ζ between facets ACE and ACD as shown in Fig. 1a,b using a small electric motor or directly folding crease AC or AB by hand. If all facets in the cellular structure are very stiff, we need to transform all units into pattern “x” together, and then transform them into the final state as shown in Fig. 1f. However, if all facets are soft (e.g. paper panels), we could switch the units one by one into their final states.

**Figure 2:**
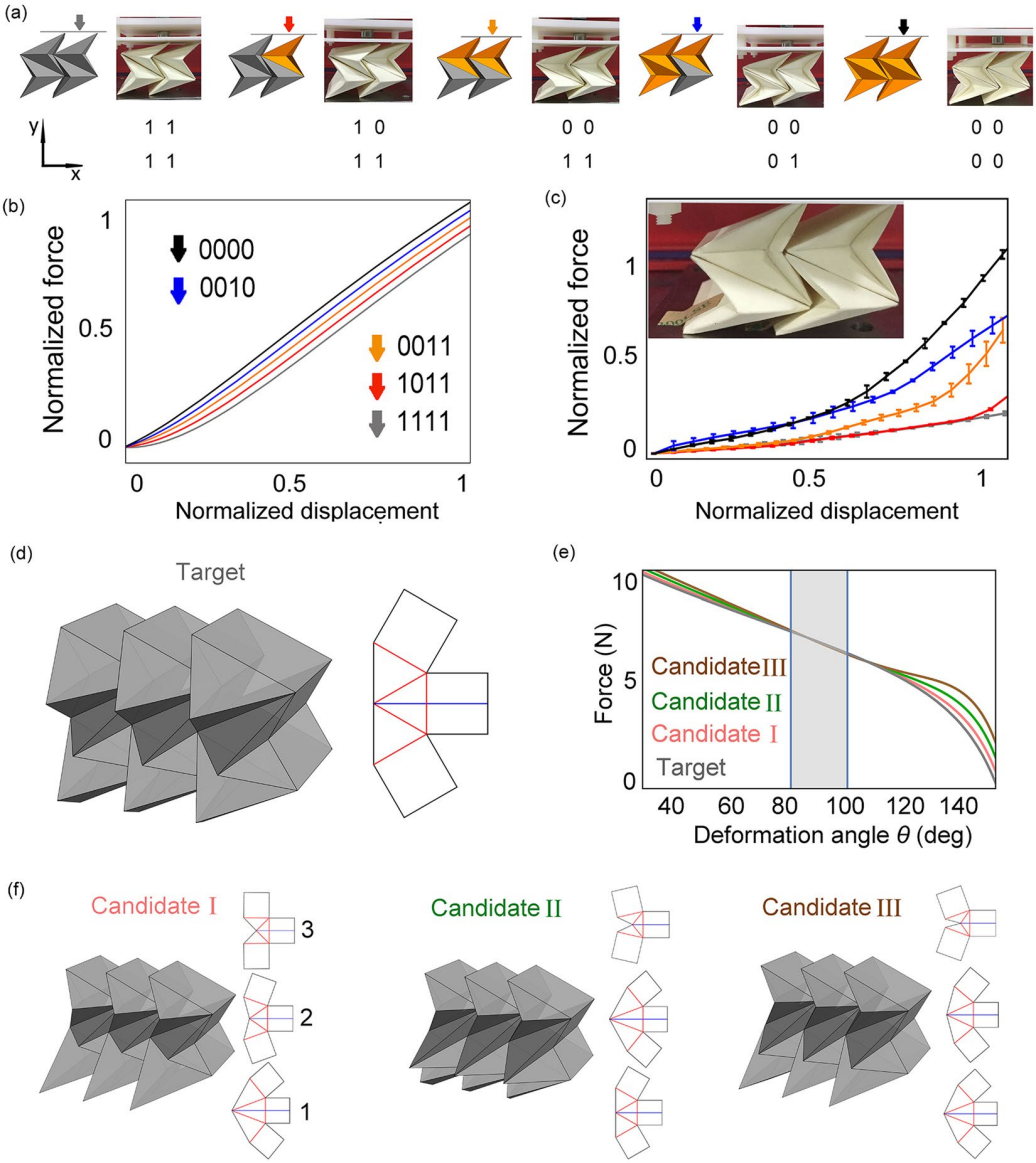
2D cellular structures comprising FO units. (**a**) 2D reprogrammable tessellation with 4 units in five patterns (simulation structures and corresponding photographs for each pattern). The arrows denote the load direction and their colors relate to (**b**) simulation curves and (**c**) experimental data, color rule: gray “1111”, red “1011”, orange “0011”, blue “0010”, black “0000”. (**d**) the target 3 × 3 tessellation and 2D folding pattern of each unit, (**e**) the force responses of the target and candidates (gray: the target structure, pink: candidate I, green: candidate II, brown: candidate III), in the gray region $(80^\circ \le \theta \le 100^\circ )$ the candidates have the same force response with the target, (**f**) the 3D configurations and 2D folding patterns of the candidates, each folding pattern corresponds to the units in each row in the tessellation, for the target: $\alpha =\beta =30^\circ$, for candidate I: ${\alpha }_{1}={\beta }_{1}=20^\circ$, ${\alpha }_{2}={\beta }_{2}=35^\circ$, ${\alpha }_{3}={\beta }_{3}=44.76^\circ$, $\sigma =0.000002$, for candidate II: ${\alpha }_{1}={\beta }_{1}=30^\circ$, ${\alpha }_{2}={\beta }_{2}=20^\circ$, ${\alpha }_{3}={\beta }_{3}=51.46^\circ$, $\sigma =0.00003$, for candidate III: ${\alpha }_{1}={\beta }_{1}=21.38^\circ$, ${\alpha }_{2}={\beta }_{2}=25^\circ$, ${\alpha }_{3}={\beta }_{3}=55^\circ$, and the error $\sigma =0.00016$ (from the bottom row to the top row in each tessellation). All units are in pattern “1”. *σ* is the error explained in SI.

Furthermore, to illustrate the multistability of the proposed origami unit, a simple but effective model is used for simulating the unit’s folding motion. By removing the facets ABD and ABE, a “virtual” spring with spring constant *k*_AB_ along AB is added in the 3D unit (see the green spring in Fig. 1g). The energy of the added spring AB can be defined as ${u}_{{\rm{AB}}}=0.5{k}_{{\rm{AB}}}{(\frac{\overline{{{\rm{AB}}}_{3{\rm{D}}}}}{\overline{{\rm{AB}}}}-1)}^{2}$, where $\overline{{{\rm{AB}}}_{3{\rm{D}}}}$ and $\overline{{\rm{AB}}}$ are the distances between points A and B in 3D unit (a function with deformation angle $\theta$, see Fig. 1h) and 2D pattern (constant, see the 2D pattern in Fig. 1a), respectively. In practice, ${u}_{{\rm{AB}}}^{\ast }$ is used as a calibration of *u*_AB_ (SI Section 3). The stretching and bending contributions of all facets are converted into the two facets ABD and ABE (which, we assume that, carries the major facet deformations). By using the virtual spring AB, the bending of facets ABD and ABE during origami folding can be qualitatively simulated. Also, a torsional spring with spring constant ${k}_{{\rm{BJ}}}$ and natural angle ${\theta }_{0}$ is introduced to model the folding motion of crease BJ^3^ with the energy of crease BJ being defined as ${u}_{{\rm{BJ}}}=0.5{k}_{{\rm{BJ}}}{(\theta -{\theta }_{0})}^{2}$. Assuming all the facets being rigid and the energies of other creases being neglected, clearly, the two added “springs” can serve as the two main drives that control the folding motion of the unit with one DOF. The energy of the unit can then be defined as $u={u}_{{\rm{AB}}}^{\ast }+{u}_{{\rm{BJ}}}$, and the normalized energy can be defined as ${u}_{n}=\frac{u}{{k}_{{\rm{BJ}}}}$. Also, we can then calculate the ratio $\overline{{{\rm{AB}}}_{3{\rm{D}}}}/\overline{{\rm{AB}}}$. As shown in Fig. 1h, $\overline{{{\rm{AB}}}_{3{\rm{D}}}}$ first decreases (the virtual spring resists it) and then increases (the virtual spring promotes it) along the axis of *θ*. Only for $\theta =0^\circ$ and $\theta =180^\circ$, there is no potential energy in the virtual spring. In Fig. 1i, the simplified model is verified by the compression experiments. It can be found that the stiffness of pattern “x” (stiff mode) is about 98.4 times as much as that of pattern “1” (soft mode), and 49.2 times as much as that of pattern “0” (soft mode) (SI Section 4). Although the curves approach the axis of zero force in Fig. 1i and it doesn’t strictly show multistability in $80^\circ  < \theta  < 120^\circ$, we could use larger value of ${k}_{{\rm{AB}}}/{k}_{{\rm{BJ}}}$ to yield multistability. Figure 1j shows that the energetic competition between the virtual spring AB and the torsional spring BJ that generates multistability (three local energetic minima), especially for large ${k}_{{\rm{AB}}}/{k}_{{\rm{BJ}}}$ (states “1”, “x”, and “0”). For the reason of the errors between the simulation and experiment in Fig. 1i, all facets are assumed as rigid panels with zero-thickness, and some experiment parameters that can sensitively influence the behavior of paper panels (e.g. temperature and humidity) are not included in the simple model.

### Reprogrammable mechanical properties of 2D tessellation made by multiple FO units

For simplicity, a 2D tessellation with 4 units in 5 patterns is designed, as shown in Fig. 2a (the simulation configurations correspond to the photograph configurations, and they correspond to the matrix with “0”s and “1”s). A uniaxial force is applied from the top of the structure, whose direction is shown as the arrows in Fig. 2a. To simulate the mechanical property of the tessellation in compression, energy differential method is used to calculate the force. Here, the calculated energy is the total energy of 4 units in each pattern (details can be found in SI Section 5). For the simulation results, Fig. 2b shows that each pattern has an individual force response, and the tessellation generates larger value of force with more “0” units. This accords with the former experiment of a unit, since the force of “0” unit is larger than that of “1” units for $\theta  < 110^\circ$ (Fig. 1i). As a direct experiment, we compress the tessellation in each pattern and obtain the similar results as shown in Fig. 2c, where the insert shows the overall experimental setup and how the tessellation is fixed with the upper and lower substrate by tapes. The sample is compressed from about 50 mm to 30 mm (height) before fully folded. Here, the normalized force (displacement) are obtained as the force (displacement) divided by its maximum. Expectably, the 1D bars have reprogrammable static and dynamic properties as shown in SI Section 6.

### Inverse design problem

Based on the force model of 2D tessellations, an interesting inverse design problem is raised: can different geometries of cellular structures produce the same force response? In essence, this is to solve an uncertain equation based on the variable design parameters and target mechanical property. To address it, we intended to find candidate 2D tessellations with different geometries but having the same force response with the target structure in a prescribed folding range by optimization. The target tessellation with 3 × 3 units is designed as a homogenous structure with $\alpha =\beta =30^\circ$ for all units, as shown in Fig. 2d. The inverse design results indicate that the optimized candidates’ behaviors approach to the target tessellation in the range of $80^\circ \le \theta \le 100^\circ$ (Fig. 2e, force curves in gray region). The candidates are three gradient structures with three identical units in each row as shown in Fig. 2f (all units are in pattern “1”, and all the geometric parameters are shown in the figure caption). See SI Section 7 for details.

### Complex 2D and 3D metastructures constructed using FO units

**Figure 3:**
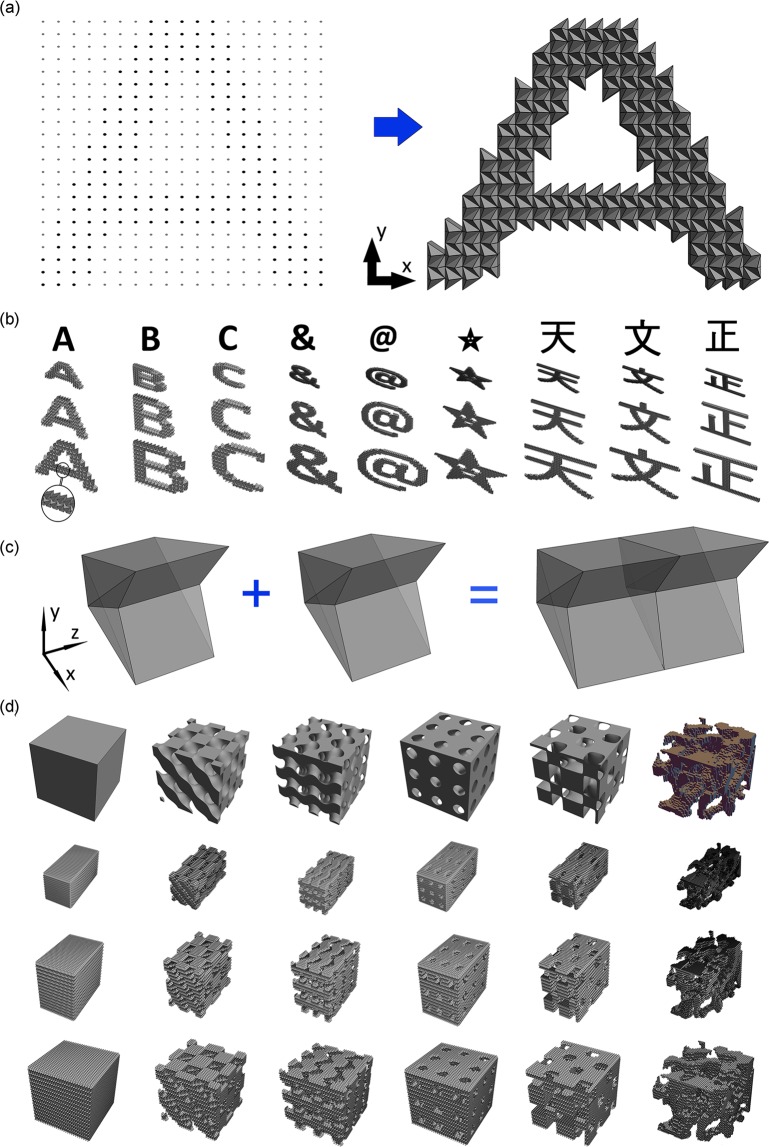
Complex 2d and 3D metastructures comprising FO units. (**a**) An example of making “A”-shaped metastructure using FO units: from binary image with pixels to 2D object, (**b**) 2D characters (from left: three English letters, two characters, a smile star and three Chinese words), (**c**) The way to construct 3D structures in Z direction, (**d**) 3D metastructures (from left: a cubic structure, four TPMSs, and a micro-CT scan of mouse femur bone), for (**b**,**d**), the 1^st^ row: target objects, the 2^nd^ row: structures with the deformation angle $\theta =60^\circ$, the 3^rd^ row: $\theta =120^\circ$, and the 4^th^ row: $\theta =180^\circ$, for all units $\alpha =\beta =60^\circ$.

To create complex structures for given applications (e.g. porous structures used in aerospace or tissue engineering), we propose a “modular” concept to combine origami-based units with inherent geometric compatibility. Figure 3a shows that the 2D object in “A”-shape can be constructed from the 2D binary image of “A” with pixels. In Fig. 3a (left), there are 22 × 19 dots forming a 2D rectangle (each dot denotes a center of pixel), and the black dots form letter “A” (other dots are in gray). The 2D “A”-shaped metastructure can be obtained by arranging the FO units into the pixels with black dots as shown in Fig. 3a (right). In this way, we simulate other 2D characters, including English letters, characters, smile star, and Chinese words, generated by FO units in Fig. 3b to show the flexibility that FO units form sophisticated structures.

Similarly, a 3D cube can be represented by *n* × *n* × *n* voxels. We arrange FO units into given voxels then create the corresponding 3D metastructures. Besides combining FO units in X-Y plane (Figs. 2 and 3a), units need to be stacked along Z direction (Fig. 3c). Figure 3d shows that 3D metastructures (*n* = 29), such as: a cube, four types of triply periodic minimal surfaces (TPMS, minimal surface is a surface with the smallest possible area for spanning given boundary, so it necessarily has zero mean curvature. TPMS is derived by periodically repeating such surfaces in three dimensions), and a micro-CT volumetric scan of femur bone part, can be constructed by FO units as voxels. For the cubic metastructures, all *n* × *n* × *n* voxels are assigned with FO units. For the TPMS-based structures, FO units should be arranged into the voxels in the region $s(i,j,k) > 0$, where $(i,j,k)$ is the coordinate of the voxel’s order number in X, Y, and Z direction $(1\le i,j,k\le n)$, and *s* is the TPMS-based function (see SI Section 8). Additionally, the femur shape is obtained by micro-CT data, which is actually a 3D binary image. Based on the same method as the 2D case (Fig. 3a), the 3D femur-inspired metastructure is formed.

With this “modular” concept, we can fabricate materials in almost arbitrary complex shapes with *N* unit cells $(N\le {n}^{3})$. Since each unit has two patterns (“0” and “1”), the whole structure has 2^*N*^ patterns. Additionally, all units can be switched into pattern “x” together due to geometrical compatibility to form a new pattern. Thus, 2^*N*^ + 1 patterns yield at most 2^*N*^ + 1 possible mechanical properties (e.g. Young’s modulus, stiffness, strength or inherent frequency).

## Conclusions

The major innovation of this research lies in two aspects: (1) a new type of origami pattern has been developed, with which the corresponding origami structure can be expanded in three directions with very few kinematic constraints. This is very useful to design programmable metamaterials, since the cellular structure can freely transform into more geometrical patterns in order to obtain more mechanical properties. (2) the inherent multistability of the origami structure is investigated, which gives rise to the re-programmability on force-displacement relation and dynamic frequency-amplitude response.

In the future, a more realistic model can be developed based on this study to simulate the bending behavior on each facet during the folding motion of a unit and the unit-unit interactions in cellular structures. Also, the mechanical behavior of structure made by different materials can further investigated.

## Materials and Methods

### Fabrication

The FO units were made by using Strathmore 500 Series 3-ply Bristol card stock which was cut by a laser cutter based on the 2D folding patterns (Fig. 1a) generated by Mathematica 10.2. The edges were taped to form the 3D units. For the 1D and 2D cellular structures, units were glued with the facets, such as facets CEIG or CDHG (Fig. 1a), using double sided tapes.

### Compression experiment

In compression measurements, force was applied to various samples at a constant loading speed of 10 mm/min. The initial heights of the unit and the 2D tessellation were about 34 mm and 51 mm, respectively (see SI Section 5 for definitions). Experiments were repeated three consecutive times for each structure and averaged. Error estimates are made with the minimum and maximum error values across all repeated measurements, demonstrating a high degree of reproducibility in the hand-made prototypes. For all tested units, $\alpha =\beta =60^\circ$ and $b=q=20\,{\rm{mm}}$.

## Supplementary information

Supplementary Material
