# Supplementary material for: Modular metamaterials composed of foldable obelisk-like units with reprogrammable mechanical behaviors based on multistability
Source: Sci Rep. 2019 Dec 11;9:18812. doi: 10.1038/s41598-019-55222-7 (PMC6906291; doi:10.1038/s41598-019-55222-7)
Supplement: Supplementary file 1 — Supplementary Material [file 41598_2019_55222_MOESM1_ESM.pdf]

## *Supporting Information*

# Modular metamaterials composed of foldable obelisk-like units with reprogrammable mechanical behaviors based on multistability

*Nan Yang*[1], *Mingkai Zhang*[2], *Rui Zhu*[2], *Xiao-dong Niu*[1]

[1] Intelligent Manufacturing Key Laboratory of Ministry of Education, Shantou University, Shantou 515063, China

[2] Key Laboratory of Dynamics and Control of Flight Vehicle, Ministry of Education, School of Aerospace Engineering, Beijing Institute of Technology, Beijing, 100081, China.

Corresponding authors:

N.Y. (nyang@stu.edu.cn), R.Z. (rzhu83ac@gmail.com), or X.D.N. (xdniu@stu.edu.cn)

## 1. Overall geometry of foldable obelisk-like (FO) unit

In the main text, we defined the dihedral angle between facets ACD and ACE as $\zeta$, and we have

$$\sin(\zeta/2) = \sin(\alpha)\sin(\theta/2)/\sin(\alpha+\beta) \tag{S1}$$

so $\Delta\zeta$ in Fig. 1c and d is defined as

$$\Delta\zeta = |\zeta^1_{\theta=180^\circ} - \zeta^0_{\theta=180^\circ}| = 2\left(\pi - 2\sin^{-1}\left(\frac{\sin(\alpha)}{\sin(\alpha+\beta)}\right)\right) \tag{S2}$$

where $\zeta^1_{\theta=180^\circ}$ and $\zeta^0_{\theta=180^\circ}$ denote angle $\zeta$ with $\theta = 180^\circ$ in pattern "1" and pattern "0", respectively.

The angle between lines GC and AC is defined as $\angle\mathrm{GCA}$, and we have

$$\angle\mathrm{GCA} = \pi/2 + \tan^{-1}(\tan(\pi/2-\beta)\cos(\zeta/2)) \tag{S3}$$

$$\angle\mathrm{ECD} = 2\sin^{-1}(\overline{\mathrm{BE}}\sin(\theta/2)/\overline{\mathrm{CE}}) \tag{S4}$$

Moreover, we define the lengths as

$$\overline{\mathrm{CE}} = b(\sin(\alpha) + \tan(\beta)\cos(\alpha))$$

$$\overline{\mathrm{BE}} = b\,\sin(\alpha);$$

$$\overline{\mathrm{AC}} = b\,\cos(\alpha)/\cos(\beta)$$

$$\overline{\mathrm{AB}} = b\cos(\alpha)$$

$$\overline{\mathrm{AB}_{3\mathrm{D}}} = \sqrt{(x_A - x_B)^2 + (y_A - y_B)^2 + (z_A - z_B)^2}$$

(S5)

Removing facets ABD and ABE, the coordinates of all points are calculated as

$$\begin{pmatrix} x_C \\ y_C \\ z_C \end{pmatrix} = \begin{pmatrix} 0 \\ 0 \\ q \end{pmatrix}, \begin{pmatrix} x_G \\ y_G \\ z_G \end{pmatrix} = \begin{pmatrix} 0 \\ 0 \\ 0 \end{pmatrix}, \begin{pmatrix} x_A \\ y_A \\ z_A \end{pmatrix}_{\text{convex}} = \begin{pmatrix} x_C \\ y_C \\ z_C \end{pmatrix} + \overline{\mathrm{AC}} \begin{pmatrix} \cos(\angle \mathrm{GCA} - \pi/2) \\ 0 \\ \sin(\angle \mathrm{GCA} - \pi/2) \end{pmatrix},$$

$$\begin{pmatrix} x_A \\ y_A \\ z_A \end{pmatrix}_{\text{concave}} = \begin{pmatrix} x_C \\ y_C \\ z_C \end{pmatrix} + \overline{\mathrm{AC}} \begin{pmatrix} \cos(\angle \mathrm{GCA} - \pi/2) \\ 0 \\ -\sin(\angle \mathrm{GCA} - \pi/2) \end{pmatrix}, \begin{pmatrix} x_E \\ y_E \\ z_E \end{pmatrix} = \begin{pmatrix} x_C \\ y_C \\ z_C \end{pmatrix} + \overline{\mathrm{CE}} \begin{pmatrix} \cos(\angle \mathrm{ECD}/2) \\ \sin(\angle \mathrm{ECD}/2) \\ 0 \end{pmatrix},$$

$$\begin{pmatrix} x_B \\ y_B \\ z_B \end{pmatrix} = \begin{pmatrix} x_E \\ y_C \\ z_E \end{pmatrix} - \overline{\mathrm{BE}} \begin{pmatrix} \cos(\theta/2) \\ 0 \\ 0 \end{pmatrix}, \begin{pmatrix} x_D \\ y_D \\ z_D \end{pmatrix} = \begin{pmatrix} x_E \\ -y_E \\ z_E \end{pmatrix}, \begin{pmatrix} x_I \\ y_I \\ z_I \end{pmatrix} = \begin{pmatrix} x_E \\ y_E \\ z_E \end{pmatrix} - \begin{pmatrix} 0 \\ 0 \\ q \end{pmatrix},$$

$$\begin{pmatrix} x_J \\ y_J \\ z_J \end{pmatrix} = \begin{pmatrix} x_B \\ y_B \\ z_B \end{pmatrix} - \begin{pmatrix} 0 \\ 0 \\ q \end{pmatrix}, \begin{pmatrix} x_H \\ y_H \\ z_H \end{pmatrix} = \begin{pmatrix} x_I \\ -y_I \\ z_I \end{pmatrix}$$

(S6)

**2. $\Delta\zeta$ : the degree of difficulty of pattern “1” switching to “0”**

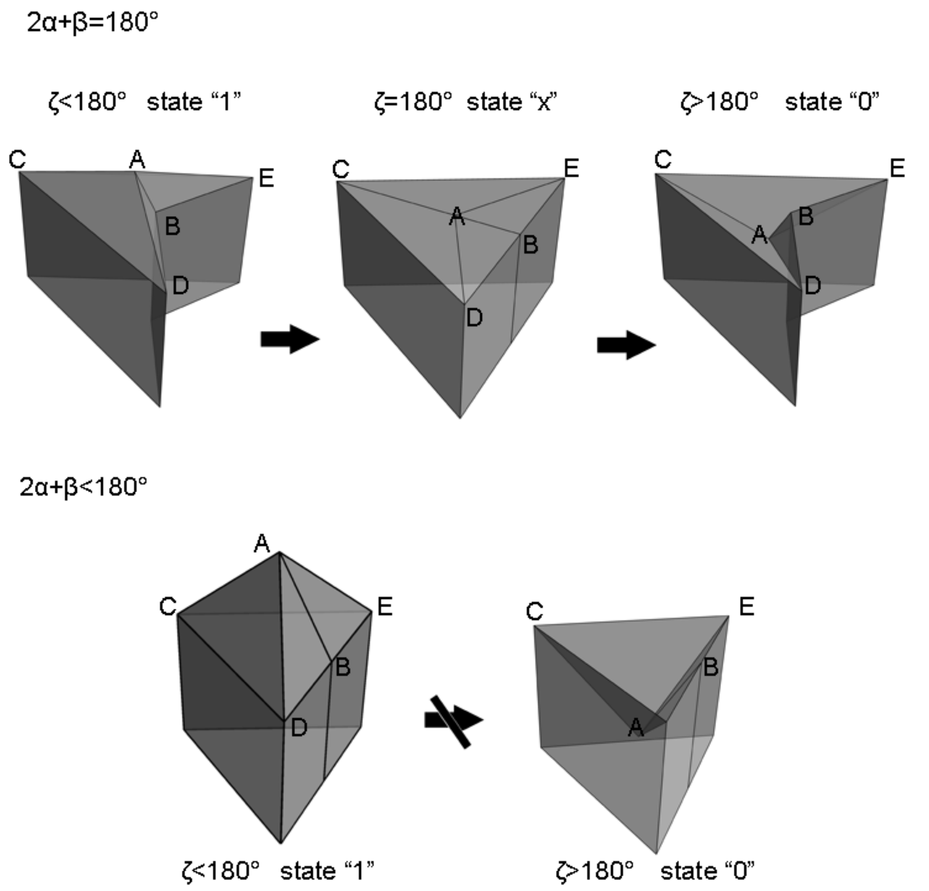


Fig. S1. The degree of difficulty that state "1" switch to state "0" for $2\alpha+\beta=180°$ and $2\alpha+\beta<180°$.

As mentioned in main text, $\Delta\zeta=|\zeta^1_{\theta=180°}-\zeta^0_{\theta=180°}|$,where $\zeta^1_{\theta=180°}$ denotes angle $\zeta$ with $\theta=180°$ in state "1", and $\zeta^0_{\theta=180°}$ denotes angle $\zeta$ with $\theta=180°$ in state "0". In Fig. S1, angle $\zeta$ is defined as the dihedral between facets DAC and EAC.

If $2\alpha+\beta=180°$ , then points A, B, C, D, E are coplanar with $\theta=180°$, and $\zeta^1_{\theta=180°}=\zeta^0_{\theta=180°}=180°$. Thus $\Delta\zeta=0$.This indicates that state "1" can smoothly switch to state "0".

However, if $2\alpha+\beta<180°$ , then we have $\zeta^1_{\theta=180°}<180°$, $\zeta^0_{\theta=180°}>180°$. Thus $\Delta\zeta>0$, and state "1" can't switch to state "0". Therefore, the physical meaning of $\Delta\zeta$ is the degree of difficulty that state "1" switch to state "0".

## 3. FO unit mechanics

When (plane angles $(\alpha,\beta)$ and lengths $(b,q)$ are fixed, we define the energy of torsional spring BJ

as ([3] in main text references)

$$u_{\mathrm{BJ}}(k_{\mathrm{BJ}}, \theta_0; \theta) = 0.5k_{\mathrm{BJ}}(\theta - \theta_0)^2 \tag{S7}$$

The energy of virtual spring AB is defined as

$$u_{\mathrm{AB}}(k_{\mathrm{AB}}; \theta) = 0.5k_{\mathrm{AB}}\left(\frac{\overline{\mathrm{AB}_{3\mathrm{D}}}}{\overline{\mathrm{AB}}} - 1\right)^2 \tag{S8}$$

But in practice, $u_{\mathrm{AB}}$ needs to be offset with a small angle $\theta^*$ as a calibration:

$$u^*_{\mathrm{AB}}(k_{\mathrm{AB}}, \theta^*; \theta) = u_{\mathrm{AB}}(k_{\mathrm{AB}}; \theta + \theta^*) \tag{S9}$$

Thus, the total energy $u$ of the unit is

$$u(k_{\mathrm{BJ}}, \theta_0, k_{\mathrm{AB}}, \theta^*; \theta) = u^*_{\mathrm{AB}}(k_{\mathrm{AB}}, \theta^*; \theta) + u_{\mathrm{BJ}}(k_{\mathrm{BJ}}, \theta_0; \theta) \tag{S10}$$

Then the normalized energy $u_n$ is defined by

$$u_n = \frac{u(k_{\mathrm{BJ}}, \theta_0, k_{\mathrm{AB}}, \theta^*; \theta)}{k_{\mathrm{BJ}}} \tag{S11}$$

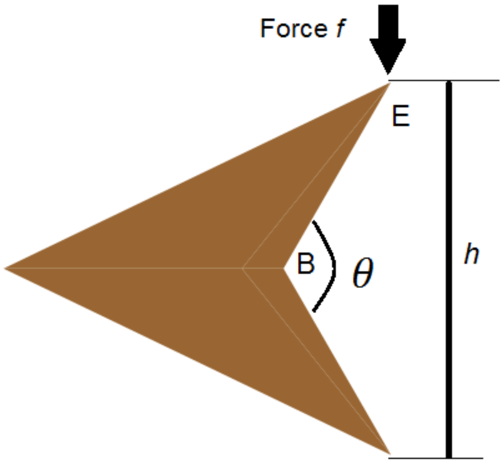


Fig. S2. Compression model of a unit.

The force $f$ is derived by the energy $u$ and the displacement as ([10] in main text references)

$$f(k_{\mathrm{BJ}}, \theta_0, k_{\mathrm{AB}}, \theta^*; \theta) = -\frac{du}{dh} = \frac{-\partial_\theta u(k_{\mathrm{BJ}}, \theta_0, k_{\mathrm{AB}}, \theta^*; \theta)}{\partial_\theta h}$$

(S12)

where $h = 2\overline{\mathrm{BE}}\sin\left(\frac{\theta}{2}\right)$

## 4. Model fitting for experimental force-compression data

We define the error $\varepsilon$ between the model and the experimental data as

$$\varepsilon(k_{\mathrm{BJ}}, \theta_0, k_{\mathrm{AB}}, \theta^*) = \sum_{i=1}^{n_{data}} \left(f(k_{\mathrm{BJ}}, \theta_0, k_{\mathrm{AB}}, \theta^*; \theta_i) - f^{exp}(\theta_i)\right)^2$$

(S13)

where $f^{exp}(\theta_i)$ is experimental force result, and $n_{data}$ is the number of the experiment data ($n_{data} = 7320$).

The parameters $(k_{\mathrm{BJ}}, \theta_0, k_{\mathrm{AB}}, \theta^*)$ can be obtained by minimizing $\varepsilon$ as

$$\min\ \varepsilon(k_{\mathrm{BJ}}, \theta_0, k_{\mathrm{AB}}, \theta^*)$$

subject to

$$k_{\mathrm{BJ}} > 0$$

$$k_{\mathrm{AB}} > 0$$

$$0 < \theta_0 < 180°$$

$$\theta^* > 0$$

(S14)

With 95% confidence interval, we obtained the best fit in Fig. 1i (main text) as: for the convex pattern (gray), $\theta^* = 19°$, $k_{\mathrm{BJ}} = 5.2 \pm 0.06\mathrm{N \cdot mm}$, $k_{\mathrm{AB}} = 56.8 \pm 0.38\mathrm{N \cdot mm}$, $\theta_0 = 153.9 \pm 0.52°$; and for the concave pattern (orange), $\theta^* = 15°$, $k_{\mathrm{BJ}} = 6.8 \pm 0.02\mathrm{N \cdot mm}$, $k_{\mathrm{AB}} = 44.4 \pm 0.13\mathrm{N \cdot mm}$, $\theta_0 = 150.5 \pm 0.12°$.

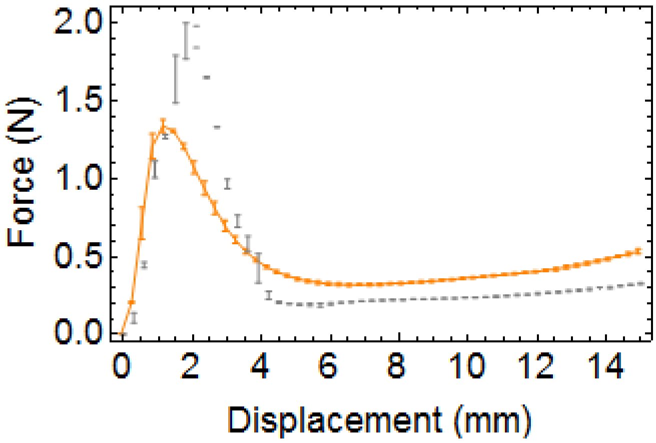


Fig. S3. Force-displacement measurements of a unit (orange: pattern “0”;gray: pattern “1”)

From the force-displacement curves (the raw data), stiffness of pattern “x” obtained from the gray unit is 1.07N/mm, and that from the orange unit is 1.49 N/mm, so the average is 1.28N/mm; stiffness of pattern “1” is 0.013N/mm; stiffness of pattern “0” is 0.026N/mm. The stiffness is obtained by the slope.

### 5. Force model of the $2 \times 2$ tessellation

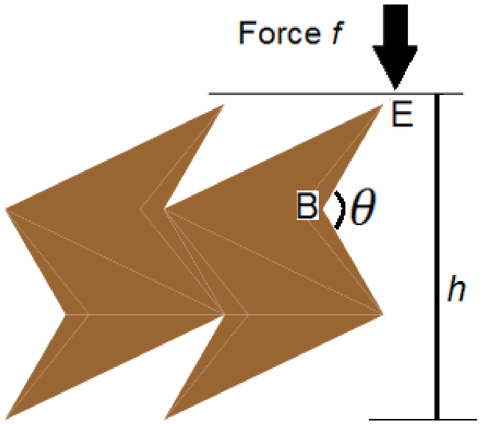


Fig. S4. Compression model of $2 \times 2$ tessellation.

The force $f_{2\times2}$ is derived by the total energy $\sum_{i=1}^{4} u_i$ and the displacement as

$$f_{2\times2}(k_{\mathrm{BJ}}, \theta_0, k_{\mathrm{AB}}, \theta^*; \theta) = -\frac{d(\sum_{i=1}^{4} u_i)}{dh} = -\frac{\sum_{i=1}^{4} \partial_\theta u_i}{\partial_\theta h}$$

where $h = 3\overline{\mathrm{BE}} \sin\left(\frac{\theta}{2}\right)$.

When there is no force applied, $h$ denotes the initial height: $h = h_0$. The energies $u_i$ of a unit in

pattern "1" and "0" are different, because the parameters $(k_{\mathrm{BJ}}, \theta_0, k_{\mathrm{AB}}, \theta^*)$ in pattern "1" and "0" are different as shown in Section 4.

## 6. Other examples of reprogrammable static and dynamic properties

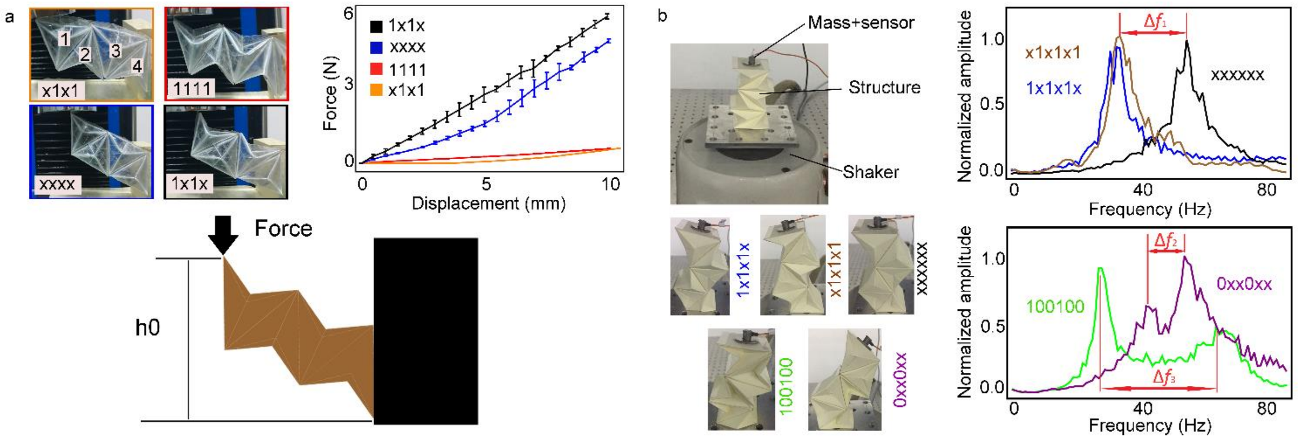


Fig. S5. (a) 1D reprogrammable bending bar with 4 units, four reprogrammed patterns (in frames of 4 colors) and the static force test data (orange frame: "x1x1", red: "1111", blue: "xxxx", black: "1x1x"), the lower figure shows the experimental setup and the definition of stress-free heights $h_0$. (b) Vibration (dynamic) experiment for the 1D bar with 6 units. In the experiment setup, the bottom of the bar is bonded on the shaker, and the top of the bar is bonded with a mass piece and an acceleration sensor. The bar in patterns "1x1x1x" (blue) and "x1x1x1" (brown) have the similar vibration response, but there is a frequency shift ($\Delta f_1 \approx 21.62$Hz) between them and pattern "xxxxxx" (black, upper plot). The interval between the two peaks of pattern "0xx0xx", $\Delta f_2 \approx 12.86$Hz (purple), changes to $\Delta f_3 \approx 37.42$Hz (green) when the bar is set in pattern "100100" (lower plot). The photographs of the five patterns are shown below the experiment setup. The vibration responses of other patterns are shown in Fig. S6.

First, we intend to design a 1D bending bar with multiple units and experimentally demonstrate its reprogrammable stiffness. By fixing its right end as a cantilever beam, we pressed the free left end and obtained four different force responses with four patterns, as shown in Fig. S5a. It is found that when fixing the pattern for No.1 and No.3 unit (counting from left), pattern "x" for No.2 and No.4 unit yield large force response (the force of "x1x1" (orange) < the force of "xxxx" (blue), and "1111" (red) < "1x1x" (black)). When fixing the pattern for No.2 and No.4 unit, pattern "1" for No.1 and

No.3 unit yield large force (“x1x1” (orange) < “1111” (red), and “xxxx” (blue) < “1x1x” (black)). Although the structural patterns “x1x1” and “1x1x” are symmetric, their force responses are quite different. This is because No.1 and No.3 unit in pattern “1x1x” need larger forces when current pattern “1” switches to pattern “x”. Since three stable states can be found for one unit (“1”, “x”, “0” ), there are in total $3^n$ ($n$ is the unit number) mechanical properties of the 1D bar. For the 1D bending bar, the stress-free heights $h_0$ of the patterns in orange, red, blue, and black frame (Fig. S5a) are 76 mm, 78 mm, 85 mm and 87 mm, respectively.

Further, we made and then tested the vibration behaviors of the 1D bar with 12 geometrical patterns (here we choose five typical patterns). Interestingly, we find that the 1D bar has the dynamic reprogrammability which can be potentially used for a dynamic low-frequency wave filter. In Fig. S5b (upper), there is a resonant frequency shift between some patterns (e.g., between “x1x1x1” (brown) and “xxxxxx” (black), $\Delta f_1 \approx 21.62\text{Hz}$) while nearly no change can be found between the “swapped” patterns (e.g., between “x1x1x1” (brown) and “1x1x1x” (blue)). In Fig. S5b (lower), the frequency interval between the two peaks can also be tuned in a programmatic fashion (e.g., for pattern “0xx0xx” (purple) the interval is $\Delta f_2 \approx 12.86\text{Hz}$; for pattern “100100” (green) the interval changes to $\Delta f_3 \approx 37.42\text{Hz}$). Each experimental result is robust as it repeats in 20 experiments.

In the dynamic study, the amplitude ratio was defined by the acceleration ratio, i.e., $\eta = \frac{a_{\text{out}}}{a_{\text{in}}}$ , where $a$ denote the amplitude and acceleration, respectively. The subscripts “out” and “in” denote the output and input, respectively. Here, $a_{\text{out}}$ comes from the acceleration sensor, and $a_{\text{in}} = 0.0002\text{m/s}^2$ in all vibration tests. The excitation signal is harmonic sinusoidal wave. In Fig. S5b, the normalized amplitude is defined as $\eta/\eta_{\text{max}}$, where $\eta_{\text{max}}$ is the maximum value in each test. The total mass of the mass piece and the acceleration sensor is about 4.0g. The mass of the 1D bar is about 6.87g. The results were averaged by 20 vibration tests. For the tested samples, $\alpha = \beta = 60°$, $b = 20\text{mm}$ and $q = 36\text{mm}$ (see main text Figure 1a for the definition of geometrical parameters). The overall vibration responses are shown in Fig. S6.

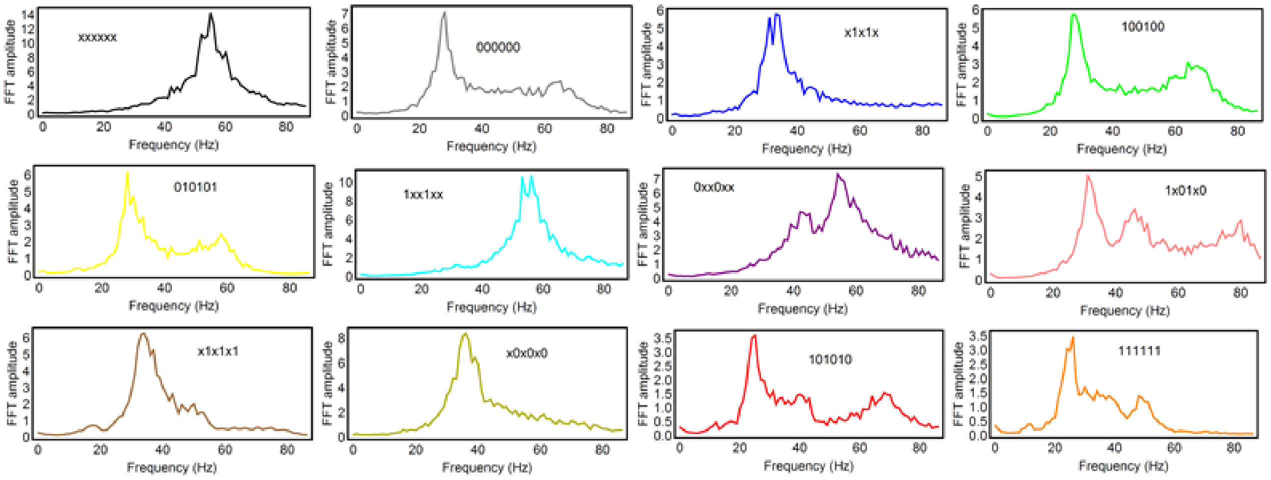


Fig. S6. Vibration responses in 12 patterns. The raw data of vibration responses in 12 programmable patterns with the same 1D bar.

## 7. Inverse design for gradient tessellations

Based on the Sections **FO unit geometry** and **FO unit mechanics**, by fixing the mechanics parameters $(k_{\mathrm{BJ}}, \theta_0, k_{\mathrm{AB}}, \theta^*)$ using the fitting results and unfixing the geometrical parameters $(\alpha, \beta)$, the energy of a unit has another form:

$$u = u(\alpha, \beta; \theta) \tag{S15}$$

When constructing gradient tessellations, the geometrical compatibility need setting the same length $\overline{\mathrm{CE}}$ as well as $\overline{\mathrm{BE}}$ for all units. Here, we set $\overline{\mathrm{CE}} = 2\overline{\mathrm{BE}} = 34.64\mathrm{mm}$ (or $\overline{\mathrm{GI}} = 2\overline{\mathrm{IJ}}$, see main text Figure 1a), then naturally we have $\alpha = \beta$, and the energy form changes to

$$u = u(\alpha; \theta) \tag{S16}$$

For the target tessellation with $3 \times 3$ units in Fig. 3a, the total energy is

$$u_{\mathrm{t}}(\theta) = 9u(30°; \theta)$$

Thus the force can be defined as

$$f_{\mathrm{t}}(\theta) = -\frac{\partial_\theta u_{\mathrm{t}}(\theta)}{\partial_\theta h} \tag{S17}$$

where $h = 4\overline{\mathrm{BE}} \sin\left(\frac{\theta}{2}\right)$

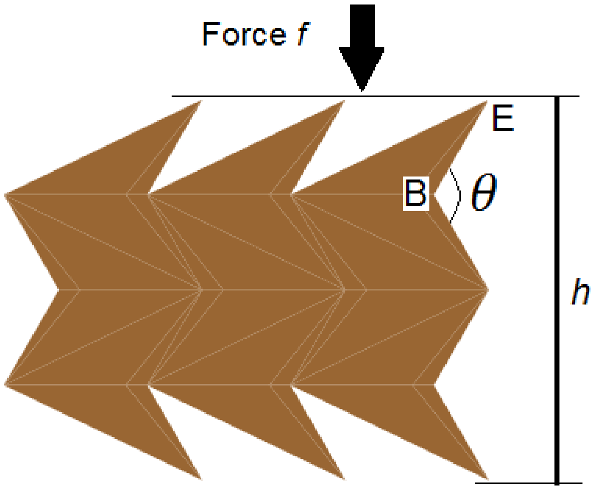


Fig. S7. Compression model of $3 \times 3$ tessellation.

For a candidate tessellation, each row was designed comprising three same units, so the energy is

$$u_{\mathrm{c}}(\alpha_1, \alpha_2, \alpha_3; \theta) = 3u(\alpha_1; \theta) + 3u(\alpha_2; \theta) + 3u(\alpha_3; \theta)$$

Thus, the force can be defined as

$$f_{\mathrm{c}}(\theta) = -\frac{\partial_\theta u_{\mathrm{c}}}{\partial_\theta h}$$

The error $\sigma$ between the candidate and target tessellation within the folding range $\theta_a \leq \theta \leq \theta_b$ is defined as

$$\sigma(\alpha_1, \alpha_2, \alpha_3) = \int_{\theta_a}^{\theta_b} (f_{\mathrm{c}}(\alpha_1, \alpha_2, \alpha_3; \theta) - f_{\mathrm{t}}(\theta))^2 d\theta \tag{S18}$$

The best solution of $(\alpha_1, \alpha_2, \alpha_3)$ is obtained by minimizing $\sigma$ as

$$\min \ \sigma(\alpha_1, \alpha_2, \alpha_3)$$

subject to

$$0 < \alpha_1 \leq 60°$$
$$0 < \alpha_2 \leq 60°$$
$$0 < \alpha_3 \leq 60° \tag{S19}$$

Since this problem has multiple solutions with the independent parameters $(\alpha_1, \alpha_2, \alpha_3)$, we can specify $\alpha_1, \alpha_2$ and find the best value for $\alpha_3$.

## 8. Construction of 3D metamaterials

First, we construct a 3D cube with $n \times n \times n$ voxels, and then assign FO units only into the voxels with $s(i,j,k) > 0$ to form a TPMS-based structure (where $(i,j,k)$ is the order number of a voxel in X, Y, and Z direction). In the main text, the four TPMSs (No.2-5 in main text Figure 3d) were defined as [1]

$$s_2(i,j,k) = \cos(wi)\cos(wj)\cos(wk) - \sin(wi)\sin(wj)\sin(wk)$$
$$s_3(i,j,k) = \sin(vi+1)\cos(vj+1) + \sin(vj+1)\cos(vk+1) + \sin(vk+1)\cos(vi+1)$$
$$s_4(i,j,k) = \sin(vi+1) + \sin(vj+1) + \sin(vk+1)$$
$$s_5(i,j,k) = \sin(wi)\sin(wj) + \sin(wj)\sin(wk) + \sin(wk)sin(wi)$$

(S20)

where the factor $w = 4\pi/n,\ v = 6\pi/n.$

## 9. Volume measurement

The fine sands with the average particle size about 38 μm were used to measure the volume of the totally deployed 3D FO units. First, we measured the density of the sands as $\rho = 1.29\text{g/ml}$, and then measured the mass $m$ of the sands that were loaded into the given unit. Finally, the full volume was calculated by $m/\rho$. The experimental results of the paper-made structures are slightly different from the numerical predictions. Fig. S8 shows that the calculated values are lower than the measured values. This is because when pouring the sands into a unit, all the facets were bended outward.

The volume $V$ is calculated by

$$V = \mathrm{S}_{\Delta\mathrm{GIH}}\left(\frac{z_\mathrm{A} - z_\mathrm{C}}{3} + q\right)$$

(S21)

where $\mathrm{S}_{\Delta\mathrm{GIH}}$ denotes the area of triangle GIH when the unit is totally unfolded (i.e., I, J and H are collinear).

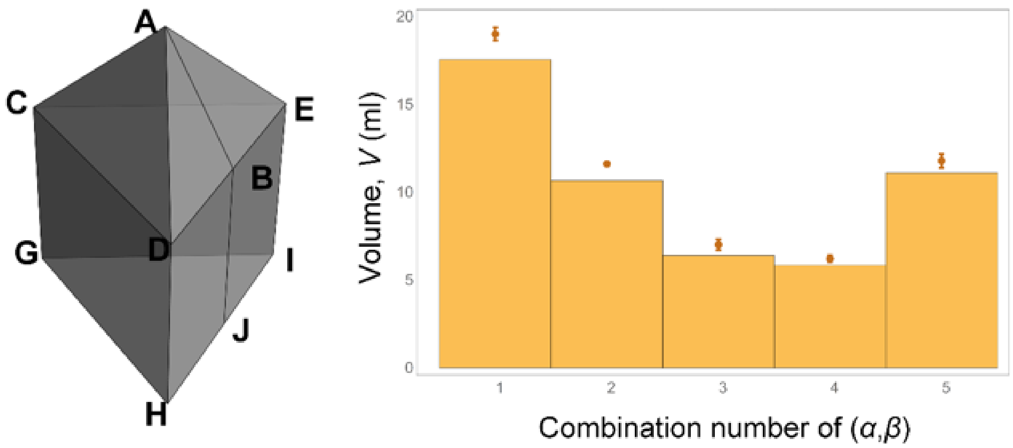


Fig. S8. The comparison between the calculated and the measured volumes with $b = q = 23$mm. 1: $(\alpha, \beta) = (40°, 60°)$, 2: $(\alpha, \beta) = (40°, 40°)$, 3: $(\alpha, \beta) = (40°, 20°)$, 4: $(\alpha, \beta) = (20°, 40°)$ ,5: $(\alpha, \beta) = (60°, 40°)$. The yellow bars show the calculated values. Error bars of the measurement data indicate the maximum and minimum results. Each structure was measured five times.

## 10. Extended 2D examples

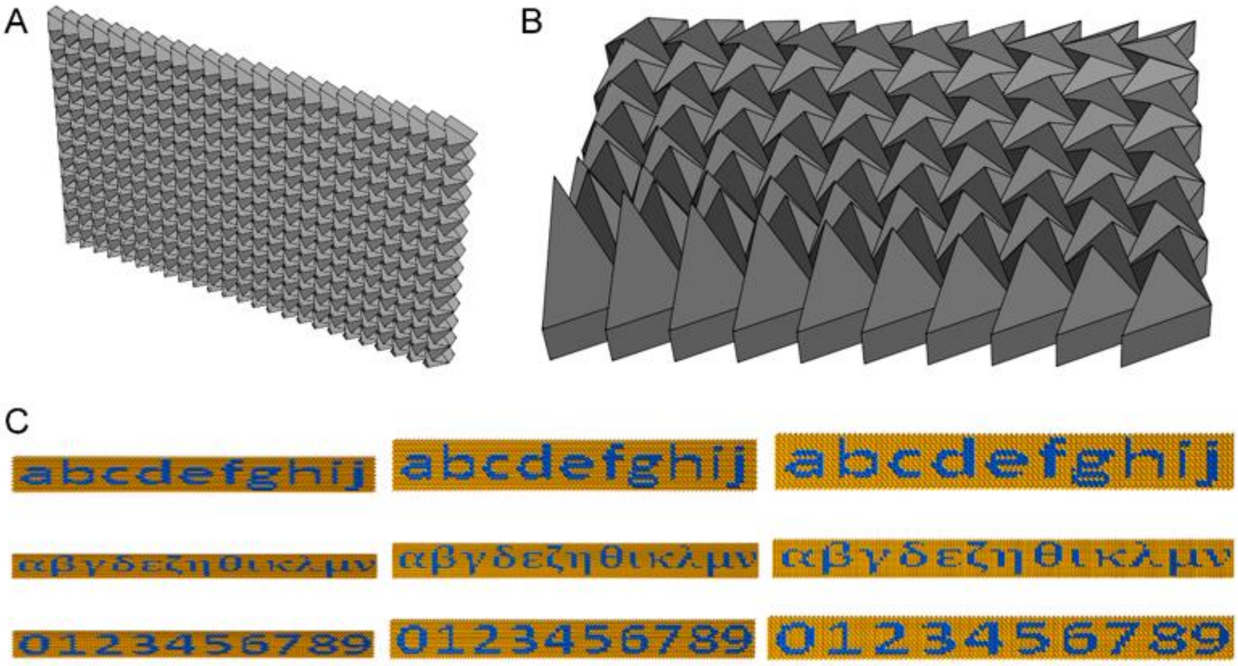


Fig. S9. Extended examples of 2D cellular structures.

(A) The 2D homogeneous structure with $25 \times 25$ units, (B) The 2D gradient structure with $10 \times 10$ units, (C) the structures programmed with English letters, Greek letters and Arabic numbers (blue units in convex pattern “1”, orange units in concave pattern “0”) with the deformation angle $\theta = 60°$, $\theta = 120°$, and $\theta = 180°$ (from left to right).

## 11. Force response of a unit fabricated using plastic sheets

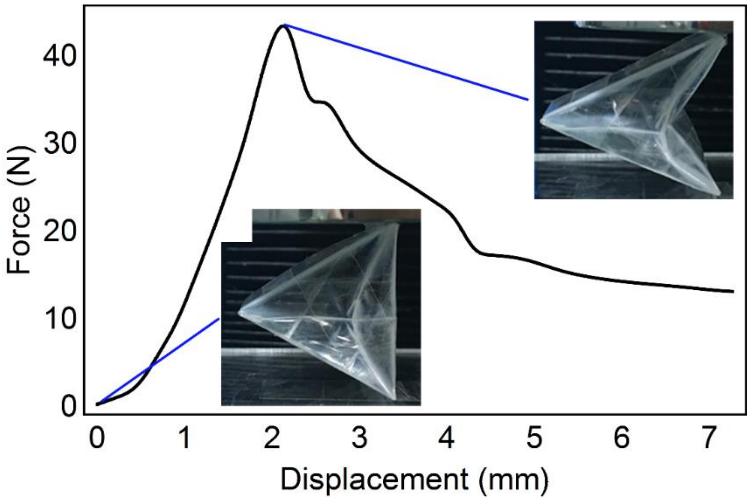


Fig. S10. Force-displacement curve of a unit fabricated using plastic sheets. We find similar negative stiffness after the compression of 2 mm.

## Reference

[1] D.J. Yoo. Porous scaffold design using the distance field and triply periodic minimal surface models. Biomaterials **32,** 7741-7754 (2011).
